# Fabrication of Injectable, Porous Hyaluronic Acid Hydrogel Based on an In-Situ Bubble-Forming Hydrogel Entrapment Process

**DOI:** 10.3390/polym12051138

**Published:** 2020-05-16

**Authors:** Lixuan Wang, Shiyan Dong, Yutong Liu, Yifan Ma, Jingjing Zhang, Zhaogang Yang, Wen Jiang, Yuan Yuan

**Affiliations:** 1 Key Laboratory for Ultrafine Materials of Ministry of Education, and School of Materials Science and Engineering, East China University of Science and Technology, Shanghai 200237, China; 18721250696@163.com (L.W.); ytl.er@foxmail.com (Y.L.); 2 Engineering Research Center for Biomedical Materials of Ministry of Education, East China University of Science and Technology, Shanghai 200237, China; 3 Department of Radiation Oncology, The University of Texas Southwestern Medical Center, Dallas, TX 75390, USA; shiyan.dong@utsouthwestern.edu; 4 Department of Chemical and Biomolecular Engineering, The Ohio State University, Columbus, OH 43210, USA; ma.1711@osu.edu (Y.M.); zhang.8211@buckeyemail.osu.edu (J.Z.)

**Keywords:** injectable hydrogel, porous structure, in-situ bubble-forming hydrogel entrapment

## Abstract

Injectable hydrogels have been widely applied in the field of regenerative medicine. However, current techniques for injectable hydrogels are facing a challenge when trying to generate a biomimetic, porous architecture that is well-acknowledged to facilitate cell behaviors. In this study, an injectable, interconnected, porous hyaluronic acid (HA) hydrogel based on an in-situ bubble self-generation and entrapment process was developed. Through an amide reaction between HA and cystamine dihydrochloride activated by EDC/NHS, CO_2_ bubbles were generated and were subsequently entrapped inside the substrate due to a rapid gelation-induced retention effect. HA hydrogels with different molecular weights and concentrations were prepared and the effects of the hydrogel precursor solution’s concentration and viscosity on the properties of hydrogels were investigated. The results showed that HA10-10 (10 wt.%, MW 100,000 Da) and HA20-2.5 (2.5 wt.%, MW 200,000 Da) exhibited desirable gelation and obvious porous structure. Moreover, HA10-10 represented a high elastic modulus (32 kPa). According to the further in vitro and in vivo studies, all the hydrogels prepared in this study show favorable biocompatibility for desirable cell behaviors and mild host response. Overall, such an in-situ hydrogel with a self-forming bubble and entrapment strategy is believed to provide a robust and versatile platform to engineer injectable hydrogels for a variety of applications in tissue engineering, regenerative medicine, and personalized therapeutics.

## 1. Introduction

In recent years, injectable biomaterials, which can be applied via minimally invasive methods in the clinic and can be formed into any desired shape to match irregular defects, have gathered much attention in the field of tissue regeneration [1,2,3,4]. In particular, injectable hydrogels, free-flowing fluids before injection that go through a spontaneous transformation into semisolid hydrogels once a reaction is initiated, have emerged as a promising platform for clinical applications. This injectable hydrogel system not only provides a biocompatible, highly hydrated 3D environment similar to the ECM structure [5,6], but can also use minimally invasive procedures to transfer cells more effectively or deliver bioactive molecules to the target site [7,8], easily filling large, irregular, and complex defects; and reducing recovery time, risk of infection, and patient pain.

From a biomimetic viewpoint, an ideal hydrogel scaffolds for tissue regeneration should contain interconnected pores to allow for effective oxygen, nutrient, and waste diffusion in a 3D environment, and cell motility, facilitating the generation of functional tissues [9]. As a matter of fact, previous studies have shown that porous hydrogels possess many advantages in 3D cell cultures over their nonporous counterparts. For example, Betz et al. [10] reported enhanced intercellular signaling of human mesenchymal stem cells in a macroporous hydrogel. Tissue engineering applications that require rapid cellular growth or expansion will benefit from the development of macroporous biohydrogels with high porosity [11,12,13,14,15]. Micron-sized interconneced pores not only promoted cell and tissue ingrowth, but also facilitated the transport of nutrients and metabolites. Meanwhile, the presence of the macropores provided the necessary space for the growth of blood vessels and thereby promoted the angiogenesis of the implant.

Given the importance of the injectability and porous structure, injectable, porous hydrogels have developed via lyophilization pore-forming, emulsion template pore-forming, porogen pore-forming, etc. Lyophilization pore-forming method [16,17,18] following in vitro injection can control the porous structure by adjusting the experimental conditions, but it is difficult to realize the possibility of applying hydrogel to minimally invasive surgery. Another emulsion template pore-forming method formed injectable porous hydrogels in situ by oil/water emulsion template technology, cross-linking by ultraviolet light, and leaching of oil droplets [19]. This method could control the pore size and porosity of the hydrogel by adjusting the oil–water ratio and the surfactant concentration. However, UV light crosslinking and paraffin oil may limit its use in vivo. Besides, leachable solid particles as sacrificial templates have been also used to create porous hydrogel constructs. Previous research mainly focused on the thiol-ene reaction among thiolated heparin, PEG-diacrylate (PEG-DA), and gelatin microparticles (GMPs). For example, by visible light, uncrosslinked GMPs quickly leached in the physiological environment, leaving the porous structure [20]. Unfortunately, many porogen pore-forming methods use cytotoxic materials as porogens, making it impossible for hydrogels to be directly applied in vivo by injection [21]. Therefore, it is strongly desirable to develop alternative, in situ pore-forming methods to fabricate injectable hydrogels. 

Hyaluronic acid (HA) has been widely used to prepare injectable hydrogels because it can mimic tissue ECM and have the potential to guide cell behavior during tissue regeneration, such as cartilage and pulp regeneration [22,23]. Moreover, the molecular structure of HA contains a large number of primary and secondary hydroxyl and carboxyl groups, so it can be cross-linked with a bifunctional small molecule cross-linking agent [24,25,26] to form a HA hydrogel [27,28,29] and can be endowed with an ideal structure, shape, hardness, and biological activity. This study endeavored to develop an injectable, porous HA hydrogel based on an in-situ, bubble-forming hydrogel entrapment process. In this experiment, an amide reaction between HA and cystamine dihydrochloride catalyzed by EDC and NHS was used to form a chemically crosslinked hydrogel. EDC and NHS generated CO_2_ bubbles during the process of activating the carboxyl group of the HA. The gelation time of the hydrogel was short enough that bubbles were unable to escape, leaving a porous structure inside the material, which achieved the combination of injectability and pore formation in situ for the hydrogels. HA hydrogels with different molecular weights and concentrations were prepared in order to study the effects of the concentration and viscosity of the hydrogel precursor solution on the injectability and porosity of the hydrogel.

## 2. Materials and Methods 

### 2.1. Materials

Hyaluronic acid (MW 100,000 Da and 200,000 Da) was purchased from Yuanye Biotechnology Co., Ltd., (Shanghai, China). Cystamine dihydrochloride was obtained from Macklin Biotechnology Co., Ltd., (Shanghai, China). 1-(3-Dimethylaminopropyl)-3-ethylcarbodiimide hydrochloride (EDC), *N*-Hydroxysuccinimide (NHS), isoamyl acetate, and sodium pentobarbital were obtained from Aladdin Industrial Co., Ltd., (Shanghai, China). Alamar Blue was bought from Maokang Biotechnology Co., Ltd., (Shanghai, China). Glutaric dialdehyde solution, fluorescein isothiocyanate-phalloidin (FITC-phalloidin), and 2-(4-amidinophenyl)-6-indolecarbamidine dihydrochloride (DAPI) were purchased from Sigma-Aldrich Co. (St. Louis, MO, USA). 

### 2.2. Preparation of HA Hydrogel

The HA hydrogels were prepared in distilled water at room temperature with cystamine dihydrochloride as the cross-linker and EDC and NHS as the catalysts. At first, HA was dissolved in distilled water. Then cystamine dihydrochloride was added as a cross-linker with a 2:1 molar ratio of carboxyl and amino groups. To this mixture, NHS and EDC were added as catalysts in a 1.5:1 molar ratio with the carboxyl group of HA. The reaction mixture was transferred to glass dishes and incubated at 37 °C for gelation. Hydrogels HA10-10 (10 wt.%, MW 100,000 Da), HA10-5 (5 wt.%, MW 100,000 Da), HA20-2.5 (2.5 wt.%, MW 200,000 Da), and HA20-1.5 (1.5 wt.%, MW 200,000 Da) were used for the experiments.

### 2.3. Rheological Measurement

Rheological behaviors of HA hydrogels were analyzed with a Rotational Rheometer (RS600, Thermo Hakke Co., Waltham, MA, USA). The gelation process was characterized on the parallel plate (diameter 20 mm) under the conditions of a time sweep at 37 °C and 1 rad/s. Gelation was monitored for 60 min by observing the changes of storage modulus (G’) and loss modulus (G’’). Storage moduli of the hydrogels as a function of frequency were characterized under the conditions of a frequency sweep at 0.1–10 rad/s and a strain of 1% at 37 °C.

### 2.4. Micro-Morphologies of the Hydrogels

In order to characterize the micro-morphologies of the hydrogels, the porous structures of the unlyophilized samples were observed using an inverted fluorescence microscope (TE2000U, Nikon Co., Tokyo, Japan) and a cryo-scanning electron microscope (Cryo-SEM) (JCM-7000, HITACHI Co., Tokyo, Japan), which has the advantage of being able to observe water-containing samples under high vacuum. The solidification of the sample was achieved by using ultra-low temperature rapid freezing. Meanwhile, the porous structures of the lyophilized samples were observed using a scanning electron microscope (SEM) (JSM-6700F, JEOL Co., Tokyo, Japan). Prior to the SEM, the samples were coated with a thin layer of gold to create a conductive surface.

In order to further quantify the sizes and porosities of the porous structures of the hydrogels, micro-CT images (Skyscan 1172, Bruker Co., Karlsruhe, Germany) were analyzed and the pore structure parameters were calculated by image processing (Version: 1.17.7.2).

### 2.5. Compression Test 

Compression performances of HA hydrogels were analyzed with a universal testing machine (AG-2000A, Shimadzu Co., Kyoto, Japan) to examine the effectw of the concentration and viscosity of the hydrogel precursor solution on the mechanical behavior of the hydrogels. The sample was made into a cylinder with a height of 8 mm and a diameter of 10 mm.

### 2.6. In Vitro Swelling and Degradation Test 

In swelling test, the hydrogels were soaked in PBS and incubated in a constant temperature oscillation box (THZ-98A, Yiheng Scientific Instrument Co., Ltd., Shanghai, China) at 37 °C, with a mild shaking motion for 21 days. The hydrogels were weighed at pre-determined time points after removal of excess water carefully. The swelling ratios of hydrogels were calculated using the following formula:

(1) $$\mathrm{Swelling}\;\mathrm{ratio}=\frac{{(W}_{\mathrm{wet}}{-W}_{\mathrm{dry}})}{W_{\mathrm{dry}}}$$

where *W_wet_* and *W_dry_* are the weight of the swollen hydrogel and the weight of the dry hydrogel, respectively.

In the degradation test, the hydrogels were soaked in Tris-HCl and incubated in a constant-temperature oscillation box at 37 °C, under mild shaking for 21 days. The hydrogels were taken at pre-determined time points and lyophilized. The lyophilized samples were weighed and compared with the initial weights of the hydrogels. The degradation rates of hydrogels were calculated using the following formula:

(2) $$\mathrm{Degradation}\;\mathrm{rate}=\frac{{(W}_{\mathrm{dry}}{-W}_{d})}{W_{\mathrm{dry}}}$$

where *W_dry_* and *W_d_* are the weight of the initial dry hydrogel and the weight of the degraded dry hydrogel, respectively.

### 2.7. In Vitro Cellular Adhesion Assay

The hydrogel samples sterilized in an autoclave (YXQ DY 250, Huaxian Medical Nuclear Instrument Co., Ltd., Shanghai, China) were placed in 24-well plates and an rat bone marrow mesenchymal stem cell (rBMSC) suspension was added to the wells from the top of the sample; next came culturing in an cell incubator (SERIES 8000W, Thermo Fisher Scientific Co., Waltham, MA, USA) at 37 °C for 24 h to detect in vitro cellular adhesion of the hydrogels. SEM and fluorescence staining were used to observe cell morphology on various hydrogels. Cells were fixed with 2.5% glutaraldehyde solution for 20 min after cultured. The samples were sequentially dehydrated in a gradient from low to high ethanol concentration (30%, 50%, 70%, 80%, 90%, 100%), and each concentration was dehydrated for 3–5 min and washed away. Then, we removed the samples from the well plate and placed each on a glass slide, added the isoamyl acetate to immerse the sample in order to fully replace the residual ethanol, and then dried it in a vacuum drying box at 37 °C overnight. Cell morphology on the hydrogels after 24 h was observed by SEM. In addition, the cells adhered on the samples were stained with FITC for 45 min at 37 °C and DAPI for 10 min at room temperature. Fluorescent images of cell morphology on the hydrogels were observed by confocal microscopy (Zeiss LSM510, A1, Nikon Co., Tokyo, Japan).

### 2.8. In Vitro Cell Proliferation Assay

This experiment used Alamar Blue detection reagents to detect cytotoxicity and cell proliferation. The main component of the detection reagent is a redox indicator. In the oxidized state, it is purple-blue and non-fluorescent, and in the reduced state, it is converted into a reduction product that shows pink or red fluorescence. During cell proliferation, the intracellular metabolic intermediates and cytochromes are in a reducing environment which can reduce the dye taken into the cells; then the dye will be released to the outside and be dissolved in the medium, so that the medium changes from non-fluorescent indigo blue to fluorescent pink.

To detect cytotoxicity and cell proliferation, the rBMSC suspension was added to the wells from the top of the scaffold, and culturing took place in a cell incubator at 37 °C for 1, 4, or 7 days. After the end of the culturing, we added Alamar blue reagent to the well plate and continued incubating in the cell incubator for 2–8 h until the color of the culture medium changed from blue to pink. Finally, the fluorescence intensity which is directly proportional to the number of viable cells was measured by a fluorescence photometer (SAFIRE, Tecan Co., Mannedorf, Switzerland). 

### 2.9. In Vivo Safety Evaluation

The animal experiment in this study was approved by the Animal Research Committee of Sixth People’s Hospital, Shanghai Jiao Tong University School of Medicine (License number: SCXK (Hu) 2018-0004), which was complied with the commonly-accepted “3Rs” and also was conducted in accordance with relevant national legislation on the use of animals for research. In order to further verify the biological safety of the hydrogels in vivo, this experiment used C57 mice as a model for subcutaneous implantation of the samples. Five mice per group were used for animal study. The specific experimental steps were as follows: 8-week-old male C57 mice were injected intraperitoneally with a 1% sodium pentobarbital solution. After the mice were anesthetized, their back hair was shaved and alcohol cotton swabs were used to disinfect the surgical site. Surgical scissors were used to make incisions of about 7 mm in length on both sides of the back of the mouse. The subcutaneous fascia was cut off and a sterilized sample with a diameter of 5 mm and a thickness of about 1 mm was implanted into the subcutaneous site of the incision. Then, the wounds were sutured and mice continued to be reared. Seven days later, mice were killed by over injection with anesthesia and the samples were removed. The sections were stained using hematoxylin and eosin (H&E) and Masson’s trichrome (MT), and then observed and photographed with an inverted microscope.

### 2.10. Statistics Analysis

Results were expressed as means ± standard deviations. All data were generated using more than three independent experiments.

## 3. Results

### 3.1. Preparation of Injectable, Porous HA Hydrogel

**Scheme 1:**
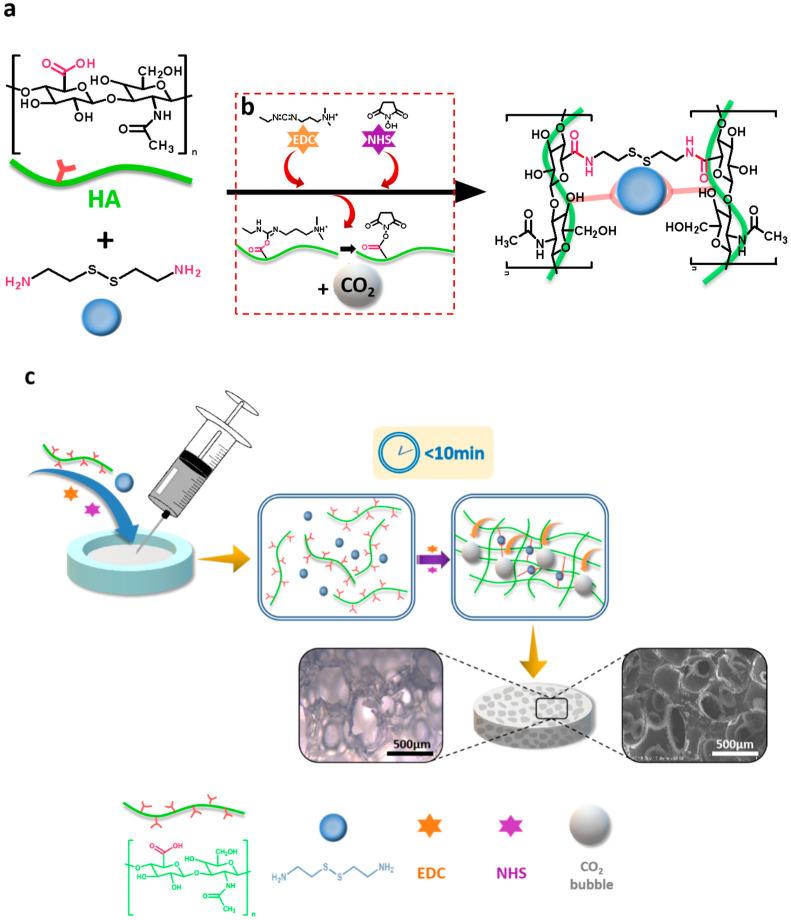
Schematic illustration of the fabrication of the injectable, porous hydrogel based on the in-situ, bubble-forming hydrogel entrapment process. (**a**) An amide reaction between HA and cystamine dihydrochloride catalyzed by EDC and NHS. Cystamine dihydrochloride was selected as a cross-linking agent to cross-link HA molecules by the reaction between carboxyl group and amino group; (**b**) EDC and NHS were used to activate the carboxyl group on HA, generating intermediate and CO_2_ bubbles; (**c**) HA hydrogel prepared in this study had a gelation time of less than 10 min. Due to the rapid gelation process, CO_2_ bubbles generated by EDC and NHS were entrapped in the 3D network, leaving a micron-sized porous structure inside the hydrogel.

As above-mentioned, to mimic the ECM that cells reside in and facilitate tissue regeneration, injectable, porous hydrogels have aroused great attention. However, successful and faithful generation of hydrogel constructs with large pore sizes after injection in vivo is still a great challenge. HA has the biological functions of integrating the extracellular matrix; promoting cell adhesion, migration, proliferation, and differentiation; and promoting angiogenesis, damage repair, and the inflammatory response to tumor metabolism. Cystamine dihydrochloride, a bifunctional cross-linking agent with a reactive primary amino group at both ends of the molecular, can undergo an amide reaction with a carboxyl group on the HA, and therefore, it was selected as a cross-linking agent to cross-link HA molecules for forming hydrogels in this study. EDC, combined with NHS, as an activator of carboxyl groups in amide synthesis, and CO_2_ bubbles, were generated during the reaction of EDC and NHS activating the carboxyl group. Moreover, the chemically cross-linked HA hydrogel in the presence of EDC and NHS was prepared in a short time, which not only satisfied the injectability of the hydrogel, but also entrapped the CO_2_ bubbles generated during the process of activating carboxyl group in the viscous hydrogel. Consequently, a porous structure was formed inside the material, thereby successfully achieving a combination of injectable and in situ pore formation. To this end, in this experiment, HA with molecular weights of 100,000 and 200,000 Da were used to prepare chemically crosslinked hydrogels. Due to the two different (in MW) forms of HA having different solubilities, the four groups of hydrogel precursor solutions have different concentrations and viscosities. Hydrogel HA10-10 (10 wt.%, MW 100,000 Da), HA10-5 (5 wt.%, MW 100,000 Da), HA20-2.5 (2.5 wt.%, MW 200,000 Da), and HA20-1.5 (1.5 wt.%, MW 200,000 Da) were prepared (Table 1) in order to study the effects of the concentration and viscosity of the hydrogel precursor solution on the injectability and porosity of the hydrogel. As shown in the Scheme 1, the hydrogel precursor solution was transferred to a syringe and injected into a mold. HA and the cross-linking agent cystamine dihydrochloride underwent a cross-linking reaction under the catalytic activation of EDC and NHS with the generation of CO_2_ bubbles. The sol-gel conversion was completed within 10 min and a porous structure was produced inside the hydrogel.

### 3.2. Characterization of the Injectability and Micro-Morphology of the Hydrogels 

**Figure 1:**
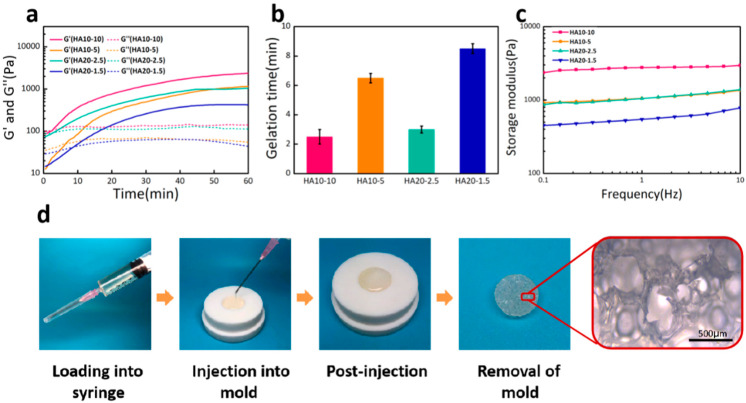
(**a**) Storage modulus (G’) and loss modulus (G’’) as a function of time for various hydrogels at 37 °C and 1 rad/s. (**b**) Gelation time of the hydrogels. HA20-1.5 had the longest gelation time about 8.5 min, whereas the gelation time of HA10-10 was much shorter about 2.5 min. (**c**) Frequency-dependent (at 37 °C and strain of 1%) oscillatory rheological analysis results. (**d**) A demonstration of the injectability and moldability of HA10-10.

In this experiment, the injectability of the HA hydrogel was evaluated by its gelation time, which was determined by measuring the time point at which storage modulus (G’) is equal to loss modulus (G’’) during time sweep. As shown in Figure 1a, the storage moduli (G’) of the hydrogels as a function of time at 37 °C, were low at initial time and started to rise with a prolonged time sweep. In the early stage, since the cross-linking reaction had not yet started, the system exhibited a resultant high liquidity, which endowed the hydrogel a desirable injectability for operation. As the reaction proceeded, a hydrogel cross-linking network started to generate, leading to an increase of G’. Based on the gelation points, the gelation times of HA10-10, HA10-5, HA20-2.5, and HA20-1.5 were 2.5, 6.5, 3, and 8.5 min, respectively (Figure 1b). It could be confirmed that the gelation times of the hydrogels were affected by the concentration and viscosity of the hydrogel precursor solutions. These hydrogels could be directly applied to tissue defects by injection due to the short gelation time. A demonstration of the injectability and moldability of HA10-10 is shown in Figure 1d.

To evaluate the response of the hydrogel to gradual loading, the frequency sweep was carried out with a frequency range of 1–10 Hz. From the curve of storage modulus with frequency, it could be seen that the four groups of hydrogels all maintained stability under gradual loading. As shown in Figure 1c, the storage moduli of HA10-10, HA10-5, HA20-2.5, and HA20-1.5 were 2955, 1358, 1381, and 778 Pa respectively, which confirms that the strengths of the hydrogels were also affected by the concentration and viscosity of the hydrogel precursor solutions.

**Figure 2:**
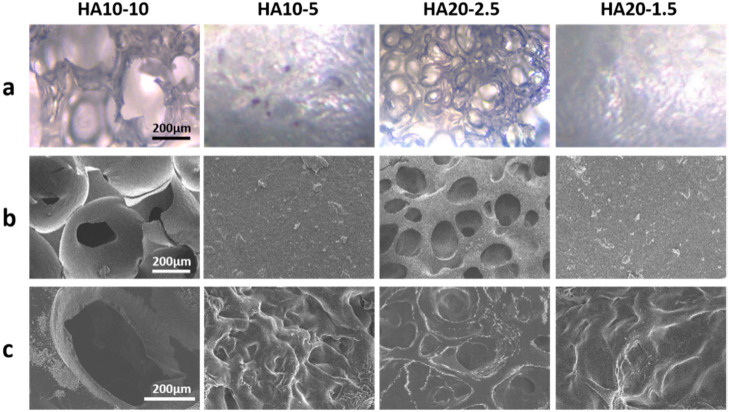
(**a**) Inverted fluorescence microscope images and (**b**) cryo-scanning electron microscope images of the hydrogels without lyophilization. (**c**) Scanning electron microscope images of the lyophilized hydrogels (scar bar: 200 μm).

The in situ porous structure formed without lyophilization was observed using an inverted fluorescence microscope and a cryo-scanning electron microscope. Both images showed that HA10-10 and HA20-2.5 had obvious porous structures and high porosity, which confirmed the feasibility of preparing an injectable, porous HA hydrogel based on the in-situ, bubble-forming hydrogel entrapment process developed in this research. The hydrogel prepared in this experiment could form a porous structure via the CO_2_ bubbles generated during the activation of the carboxyl group by EDC and NHS being entrapped in the hydrogel, and thus leaving pores inside the material. This process without lyophilization provided more possibilities for subsequent applications. As shown in Figure 2a,b, both HA10-10 and HA20-2.5 had micron-sized porous structures that are more ideal for cell migration and proliferation compared to nano-sized porous structure typically found in injectable hydrogels. The pore size of HA10-10 (400–500 μm) was larger than that of HA20-2.5 (100–200 μm), while HA10-5 and HA20-1.5 did not have obvious porous structures. These results indicated that the concentration and viscosity of the hydrogel precursor solution affected the pore size of the hydrogel. In addition, proper porosity is an important biomaterial design standard for scaffolds used in tissue engineering applications [30,31,32,33], as it can increase the cell adhesion, migration, proliferation, and extracellular matrix production of scaffolds at tissue defect sites. Therefore, the in-situ pore-forming hydrogel with micron-sized porous structure and higher porosity prepared in this experiment had excellent application prospects in the field of tissue engineering.

In addition, the microscopic morphology of the lyophilized hydrogel was observed by the scanning electron microscope. Similarly to the images of inverted fluorescence microscope and cryo-scanning electron microscope, HA10-10 and HA20-2.5 had obvious micron-sized porous structures, and the pore size of HA10-10 was larger than that of HA20-2.5. Compared to the cryo-scanning electron microscope images of the hydrogels without lyophilization, the surfaces of HA10-5 and HA20-1.5 became uneven after lyophilization (Figure 2c).

**Figure 3:**
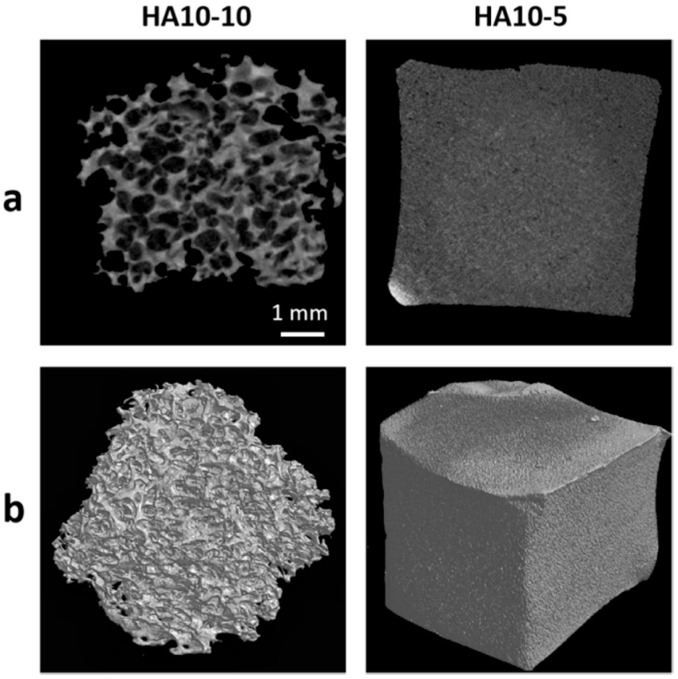
(**a**) 2D micro-CT images of the HA hydrogels; (**b**) 3D micro-CT images of the HA hydrogels. (scar bar: 1 mm).

In order to further quantify the pore structure parameters, micro-CT tests were performed on HA10-10 and HA10-5. The micro-CT image showed that HA10-10 had an interconnected, micron-level macropore structure and the average pore size was 457 μm. In contrast, the surface of HA10-5 was almost smooth, without an obvious pore structure (Figure 3). In addition, the porosity of HA10-10 exceeded 80% (Table 2), confirming a high porous structure of HA10-10 prepared in this experiment, which was conducive to the inward growth of cells and tissues.

### 3.3. Mechanical Properties, Swelling Ratios, and Degradation Rates of the Hydrogels

Matrix stiffness affects the phenotype and differentiation pathway of mesenchymal stem cells (MSCs) [34]. More and more studies show that the mechanical properties of materials have a great influence on cell behavior [35,36,37]. Two-dimensional materials and three-dimensional materials with different elastic moduli can promote the differentiation of MSCs in different directions. MSCs differentiate towards nerve cells on the surfaces of materials with low elastic moduli (0.1–1 kPa), differentiate on the surfaces of materials with medium elastic moduli (8–17 kPa) towards myoblasts, and differentiate on the surfaces of materials with high elastic moduli (34 kPa) towards osteoblasts. The hydrogel material has a three-dimensional network structure and high water content, and the mechanical environment is closer to that of the organism, which is conducive to cell growth. The elastic modulus, which is the key mechanical property of the hydrogel, is the main basis for judging the effect of cell culture substrates on cell behavior in vitro. 

**Figure 4:**
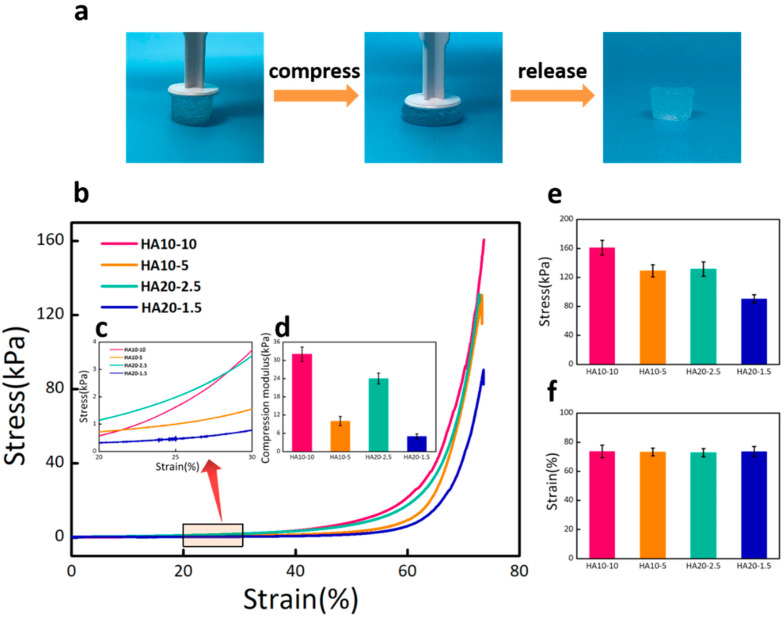
(**a**) Optical images of the hydrogel under excessive compression. (**b**) Stress–strain curves of HA10-10, HA10-5, HA20-2.5, and HA20-1.5 hydrogels under compression. (**c**) An elastic interval with the strain range of 20–30%. (**d**) Compression moduli of the hydrogels calculated from the stress–strain curve. (**e**) Compression stress and (**f**) compression strain of the hydrogels. HA10-10 had the largest compression stress about 160 kPa. There was no significant difference in compression strain of the four groups of hydrogels.

The compression test was used to characterize the compression moduli of HA hydrogels. As shown in Figure 4a, the hydrogel exhibited significant stability and almost completely recovered after excessive compression. The stress–strain curve reflected the deformation abilities of the hydrogels under stress. From the curve change trend in the Figure 4b, it can be seen that the deformations of the four groups of hydrogels gradually increased as stress increased. Under the same stress state, the greater the deformation of the hydrogel, the stronger the viscosity and the weaker the elasticity. The different deformation degrees of the four groups of hydrogels confirmed that HA10-10 had the strongest elasticity, while HA20-1.5 had the weakest elasticity. An elastic interval with the strain range of 20–30% (Figure 4c) was selected from the stress–strain curve to calculate the compression moduli of the hydrogels. The result showed that the compression modulus of HA10-10 (32 kPa) was in the range of the modulus for the differentiation of MSCs towards osteoblasts (Figure 4d). Both the compression modulus and the maximal compression stress were affected by the concentration and viscosity of the hydrogel precursor solution (Figure 4e,f).

**Figure 5:**
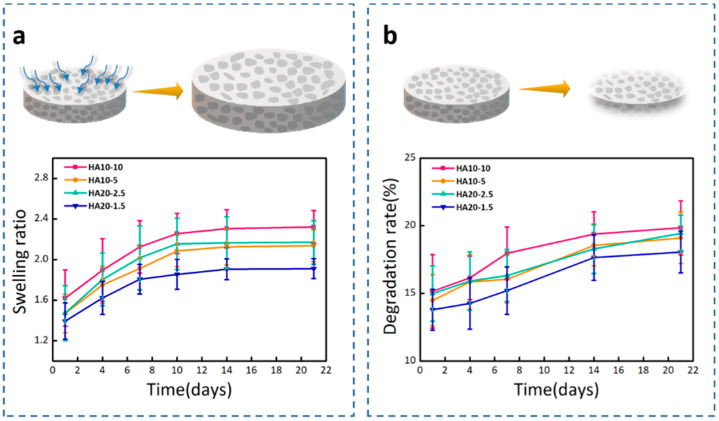
(**a**) Swelling ratios of the hydrogels in PBS (pH = 7.4). (**b**) Degradation rates of the hydrogels in Tris-HCl (pH = 7.4).

Hydrogels are hydrophilic, and can swell to an equilibrium volume in water while still maintaining their shapes and three-dimensional spatial network structures. Figure 5a showed the swelling ratios of the four groups of hydrogels in PBS (pH = 7.4). HA10-10 had the highest swelling ratio, about 2.3, which shows that the swelling ratio was improved by the porous structure of the hydrogel. In porous hydrogels, the scale criteria that determine the swelling process are pore size and porosity. The porous structure can greatly increase the swelling ratio of the hydrogel [38]; therefore, HA10-10 and HA20-2.5 with obvious porous structures and higher porosity, had larger swelling ratios compared to HA10-5 and HA20-1.5. After 7 days, the swelling ratios of the four groups of hydrogels slowed down and reached swelling equilibrium at 21 days.

In vitro degradation behavior of hydrogels using Tris-HCl (pH = 7.4) as the degradation solution was monitored at 37 °C by a weight loss method. In the degradation test, the initial degradation behavior mediated through the surface corrosion process was obvious, and after that, the weight of the hydrogel sample steadily decreased. As shown in Figure 5b, it was clearly observed that there were differences between the degradation rates of the hydrogels in Tris-HCl. On the 21st day, the degradation rates of HA10-10, HA10-5, HA20-2.5, and HA20-1.5 were 19.85%, 19.10%, 19.44%, and 18.05%, respectively, which implies that the degradation rate was also affected by the porous structure of the hydrogel. HA10-5 and HA20-1.5 did not have obvious porous structures, and the degradation behaviors were slower compared to HA10-10 and HA20-2.5.

### 3.4. Cell Adhesion, Morphology, and Proliferation of rBMSCs on the Hydrogels

**Figure 6:**
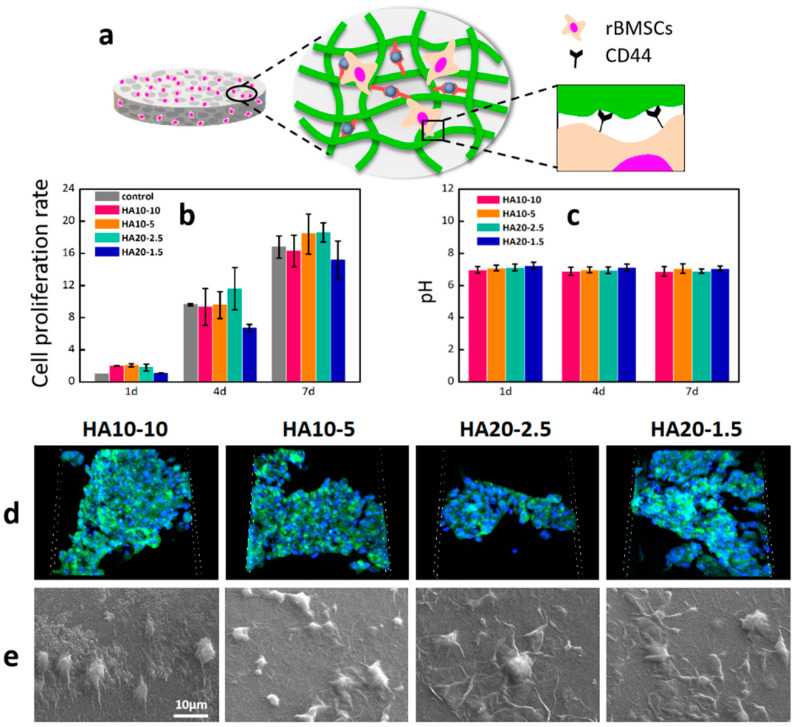
Cell adhesion, morphology, and proliferation of rBMSCs on the hydrogels. (**a**) A demonstration of rBMSCs adhesion on the surface of a HA hydrogel. (**b**) The cell proliferation on the hydrogels after culturing for 1, 4, and 7 days was further determined by Alamar Blue detection reagents; (**c**) pHs of PBS soaked in the hydrogels; (**d**) fluorescent images of cell adhesion on the hydrogels by confocal microscope with FITC and DAPI staining after 24 h (scanning box: 212 × 212 × 36 μm^3^). (**e**) Cell morphologies of the hydrogels after 24 h.

HA can bind to CD44, an important HA receptor on the cell surface, to activate CD44 and regulate various biological behaviors of cells, including cell adhesion, migration, proliferation, and differentiation [39]. In order to detect the cytotoxicity and cell proliferation of rBMSCs on HA hydrogels, the content of viable cells in the wells was determined using Alamar blue at 1, 4, and 7days respectively. As shown in Figure 6b, compared with the control group, the cell viabilities of the four groups of experimental materials were all greater than 100% on the first day, indicating that these four components could support cell adhesion and had a certain degree of cell proliferation in the short term. On the fourth day, the number of living cells on the four groups of hydrogels increased significantly compared with the first day, and the cell proliferation rates were close to those of the control group. After 7 days of culture, the cell proliferation rates of the four experimental groups reached more than 15 times, indicating that the hydrogels had good cell compatibility and the cells could proliferate well on the materials. In addition, the hydrogels were soaked in PBS and incubated in a constant-temperature box at 37 °C for 7 days. The pH values of the solutions were measured to characterize the effects of the hydrogels on the pHs of the solutions. As shown in Figure 6c, the pH values of the PBS were relatively stable at 1, 4, and 7 days.

In order to further investigate the cell behaviors on the material, the adhesion of rBMSCs after 24 h in culture was first investigated with a confocal microscope. Significant cell adhesion was observed on the materials (Figure 6d); high cell density demonstrated that HA hydrogels prepared in this study with better cytocompatibility and cell adhesive properties. The morphology of rBMSC on the surface of the material was further observed by scanning electron microscope. The cells were connected to the surface of the HA hydrogels through extended pseudopod (Figure 6e).

### 3.5. Biocompatibility of the Hydrogels

**Figure 7:**
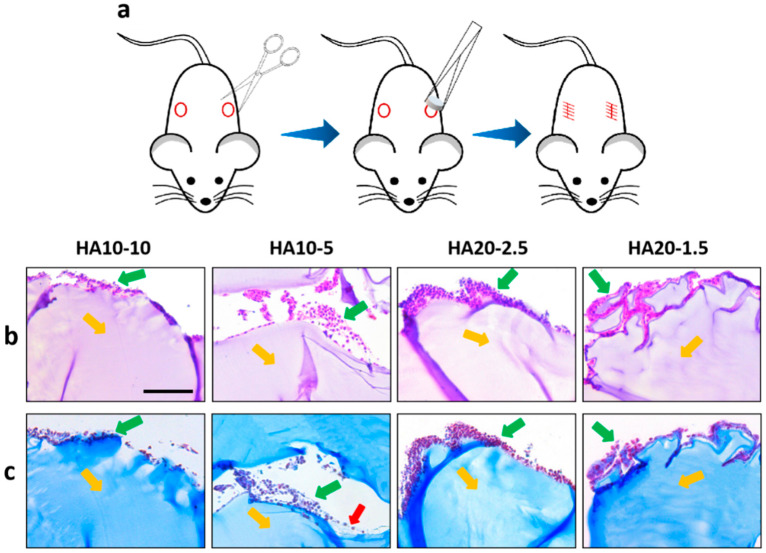
(**a**) A subcutaneous implant model in the backs of C57 mice. Inverted fluorescence microscope images of the sections stained using hematoxylin and eosin (H&E) (**b**) and Masson’s trichrome (MT) (**c**) at 7 days. Yellow arrows: implanted hydrogels, green arrows: inflammatory cells, red arrows: collagen fibers. (scar bar: 100 μm).

Due to the large differences between in vivo and in vitro environments, a one-week in vivo safety evaluation of the HA hydrogels was performed using a subcutaneous implant model in the back of C57 mice (Figure 7a). Hydrogels were implanted subcutaneously in C57 mice; the mice grew well without any discomfort. After 7 days, samples were taken out from the mice and HE/MT stained for inflammation observation. HE and MT staining showed that the inflammatory cells infiltrated around the implanted material, and the number of HA10-10 inflammatory cells infiltrated was less than for the other three groups. In addition, a few collagen fibers were observed around HA10-5 by MT staining (Figure 7b,c). In general, the HA hydrogel-induced inflammatory responses will not cause greater toxicity in vivo and could be acceptable, confirming good biocompatibility.

## 4. Conclusions

We have prepared injectable, porous chemically cross-linked HA hydrogels based on an in-situ, bubble-forming hydrogel entrapment process. The concentration and viscosity of the hydrogel precursor solution directly affected the gelation time and the amount of CO_2_ bubbles generated, and indirectly affected the formation of porous structure in the hydrogel. The rheological measurement results and micro-morphologies of the hydrogels indicated that HA10-10 and HA20-2.5 had shorter gelation times and produced obvious porous structures, which made it possible to combine the injectability with pore formation in situ of the hydrogel. Compression experiments confirmed that HA10-10 had a compression modulus that facilitates the differentiation of MSCs into osteoblasts. The swelling ratios and degradation rates of the four groups of hydrogels in vitro were affected by their porous structures. In addition, cell proliferation and adhesion assay confirmed that the HA hydrogels prepared in this experiment had good cell compatibility. In particular, HA10-10 showed the best biocompatibility. It is anticipated that our strategy utilizing in-situ, bubble-forming hydrogel entrapment could be widely extended to various tissue engineering constructs in a tissue-specific manner.

## Figures and Tables

**Table 1:** Formulation and naming of HA hydrogels.

Sample Codes	MW (Da)	Concentration (wt.%)
HA10-10	100,000	10
HA10-5	100,000	5
HA20-2.5	200,000	2.5
HA20-1.5	200,000	1.5

**Table 2:** The pore structure parameters of HA10-10.

Sample Code	Closed Porosity (%)	Open Porosity (%)	Total Porosity (%)	Average Pore Size (μm)
HA10-10	0.00227 ± 0.00081	82.93863 ± 3.14905	82.93901 ± 3.52803	457 ± 34
